# Ophthalmic Artery Chemosurgery for Less Advanced Intraocular Retinoblastoma: Five Year Review

**DOI:** 10.1371/journal.pone.0034120

**Published:** 2012-04-24

**Authors:** David H. Abramson, Brian P. Marr, Scott E. Brodie, Ira Dunkel, Sotiria Palioura, Y. Pierre Gobin

**Affiliations:** 1 Ophthalmic Oncology Service, Memorial Sloan-Kettering Cancer Center, New York, New York, United States of America; 2 Department of Ophthalmology, Mount Sinai School of Medicine, New York, New York, United States of America; 3 Department of Pediatrics, Memorial Sloan-Kettering Cancer Center, New York, New York, United States of America; 4 Department of Ophthalmology, Massachusetts Eye and Ear Infirmary, Boston, Massachusetts, United States of America; 5 Departments of Radiology and Neurosurgery, Weill Cornell Medical College, New York, New York, United States of America; University of Leipzig, Germany

## Abstract

**Background:**

Ophthalmic artery chemosurgery (OAC) for retinoblastoma was introduced by us 5 years ago for advanced intraocular retinoblastoma. Because the success was higher than with existing alternatives and systemic side effects limited we have now treated less advanced intraocular retinoblastoma (Reese-Ellsworth (RE) I-III and International Classification Retinoblastoma (ICRB) B and C).

**Methodology/Principal Findings:**

Retrospective review of 5 year experience in eyes with Reese Ellsworth (Table 1) I (7 eyes), II (6 eyes) or III (6 eyes) and/or International Classification (Table 2) B (19 eyes) and C (11 eyes) treated with OAC (melphalan with or without topotecan) introduced directly into the ophthalmic artery. Patient survival was 100%. Ocular event-free survival was 100% for Reese-Ellsworth Groups I, II and III (and 96% for ICRB B and C) at a median of 16 months follow-up. One ICRB Group C (Reese-Ellsworth Vb) eye could not be treated on the second attempt for technical reasons and was therefore enucleated. No patient required a port and only one patient required transfusion of blood products. The electroretinogram (ERG) was unchanged or improved in 14/19 eyes.

**Conclusions/Significance:**

Ophthalmic artery chemosurgery for retinoblastoma that was Reese-Ellsworth I, II and III (or International Classification B or C) was associated with high success (100% of treatable eyes were retained) and limited toxicity with results that equal or exceed conventional therapy with less toxicity.

## Introduction

Ophthalmic artery chemosurgery (OAC) for retinoblastoma was first performed 5 years ago by us as in the hope of saving eyes with extensive intraocular retinoblastoma scheduled for enucleation [1]. It has now been performed successfully in 26 countries worldwide and more than 20 peer-reviewed publications have demonstrated that the majority of eyes so treated can avoid enucleation or radiation. This has been accomplished with minimal systemic toxicity, in as few as one cycle of a single drug and with acceptable local ophthalmic toxicity.

The first attempt to deliver high doses to the eye while minimizing systemic exposure was performed by Reese more than 50 years ago with a very different rationale in mind [2]. Reese delivered intra-arterial Triethylene Melanamine (TEM) (a nitrogen mustard derivative) via direct carotid artery puncture on the side to be treated in an attempt to lower the dose of therapeutic radiation used to treat retinoblastoma. Intrarterial TEM allowed him to lower the dose by 50% (from 15,000cGy to 7,500cGy) and he wrote “it is amazing to see the clinical regression of a lesion following employment of x-ray together with one injection of TEM by way of the carotid artery” [2].

Investigators from Japan then began a different form of intra-arterial chemotherapy in retinoblastoma for a very different reason. They developed a balloon catheter that was introduced via the femoral artery and allowed for occlusion of the internal carotid artery on the side to be treated with rapid infusion of drugs (usually single agent melphalan) proximal to the balloon-hence they called it “selective intrarterial chemotherapy” [3]. They were not trying to lower the dose of radiation. In Japan clinicians faced a cultural challenge. Families of children with unilateral retinoblastoma (who could be cured of disease with just an enucleation) refused curative enucleation for cultural reasons. The physicians therefore decided that they would treat eyes with everything possible in the hope of salvaging a life by saving an eye-no matter what the ocular consequences might be. They combined their intra-arterial technique with external beam irradiation, hyperthermia, intravitreal injection of chemotherapy and focal laser and/or cryotherapy in an attempt to salvage the eye. Their recent report highlighted their success in avoiding enucleation of advanced eyes and is reassuring that no long term consequences (since 1986)-especially second cancers-were observed [4].

We introduced the technique of *super* selective infusion by advancing a micro-catheter into the orifice of the ophthalmic artery on the side to be treated (or both sides in the same session in cases of bilateral retinoblastoma- “tandem therapy” [5]) after introduction of the catheter via the femoral artery. In our initial report we demonstrated that 7 of 9 eyes scheduled for enucleation could be spared enucleation as a result of this new approach [1].

Since that first report three years ago many have reported the success and complications of this technique in (very) advanced eyes-usually scheduled for enucleation. The largest report to date is from our center. Of 95 eyes treated over a four- year period 87 were advanced (Reese-Ellsworth (RE) Groups IV–V) and only 8 less advanced eyes were treated [6]. 100% of the eyes treated in Miami were advanced (RE Vb or International Classification (ICRB) Group D) [7]. Similarly 100% of the eyes treated in Switzerland were advanced [8]. Of 17 eyes treated in Philadelphia all but 2 were similarly advanced [9].

Because our systemic toxicity in treating children with advanced intraocular retinoblastoma was limited (most frequently asymptomatic Grade 3 neutropenia), we began treating less advanced retinoblastoma and now report on the success and adverse events in these patients.

## Methods

We have previously described our technique of ophthalmic artery chemosurgery that achieves super selective delivery of chemotherapy in the eyes of children with retinoblastoma [1], [3], [10]. A 4-French arterial sheath is introduced into the femoral artery (alternating sides with each successive treatment) under general anesthesia and anticoagulation attained with intravenous heparin (75I U/kg). Microcatheters are then passed into the ophthalmic artery on the side to be treated using fluoroscopy and roadmapping. Both flow-directed catheters (such as the Magic-Balt Therapeutics, Montmorency, France) with outer diameters of 400 or 500 microns and guidewire-directed catheters (such as the Excelsior SL 10 Stryker, Freemont CA) with an outer diameter of 570 microns have been used. The chemotherapy drugs are then diluted with saline in a 30cc solution, injected in a pulsatile fashion over 30 minutes. At the end of the procedure the catheter is withdrawn, the femoral puncture site is compressed for hemostasis and the child discharged the same day. Children had either RE Groups I, II or III and/or ICRB B or C (Table 3). Although both RE Groups I-III and ICRB Groups B and C are considered less advanced groups each Group uses different criteria so the actual number of cases in RE I-III are not the same as ICRB B and C. In particular ICRB C include eyes with vitreous seeding while in RE, by definition, no seeding is present in Groups I, II or III.

Examinations under anesthesia were performed every 3 or 4 weeks and included assessment of vision, motility and pupillary responses before dilation (or anesthesia) and then after dilation (under anesthesia) an ophthalmic exam including fundus photography with the RetCam, ultrasound and retinal function monitoring with our modified full electroretinogram protocol performed (including photopic and scotopic responses) [11]. As in earlier manuscripts we report 30-Hz flicker responses as a surrogate for ERG responses. Changes in 30-Hz ERG amplitudes of less than 25 µV were considered inconsequential. Fluorescein angiograms were not done on a routine basis.

This study was performed with the approval of the Institutional Review Board at Memorial Sloan-Kettering Cancer Center. Written, informed consent (from parents) was obtained for all participants in this study.

## Results

The demographic data of the patient population are presented in Table 3.

**Table 1:** Reese-Ellsworth (RE) Classification Scheme.

Reese-Ellsworth (RE) Classification For Intraocular Retinoblastoma	
**GROUP I**	***a.*** ** Solitary tumor, less than 4 disc diameters in size, at or behind the equator**
	***b.*** ** Multiple tumors, none over 4 disc diameters in size, all at or behind the equator**
**GROUP II**	***a.*** ** Solitary tumor, less than 4 to 10 disc diameters in size, at or behind the equator**
	***b.*** ** Multiple tumors, none over 4 to 10 disc diameters in size, all at or behind the equator**
**GROUP III**	***a.*** ** Any lesion anterior to the equator**
	***b.*** ** Solitary tumors larger than 10 disc diameters behind the equator**
**GROUP IV**	***a.*** ** Multiple tumors, some larger than 10 disc diameters**
	***b.*** ** Any lesion extending anteriorly to the ora serrata**
**GROUP V**	***a*** **. Massive tumors involving over half the retina**
	***b*** **. Vitreous seeding**

**Table 2:** International Classification for Retinoblastoma (ICRB) Scheme.

International Classification for Intraocular Retinoblastoma (ICRB)	
**Group A**	*Small intraretinal tumors away from foveola and disc*
	* All tumors are 3 mm or smaller in greatest dimension, confined to the retina ***and*** * All tumors are located further than 3 mm from the foveola and 1.5 mm from the optic disc
**Group B**	*All remaining discrete tumors confined to the retina*
	* All other tumors confined to the retina not in Group A * Tumor-associated subretinal fluid less than 3 mm from the tumor with no subretinal seeding
**Group C**	*Discrete Local disease with minimal subretinal or vitreous seeding*
	* Tumor(s) are discrete * Subretinal fluid, present or past, without seeding involving up to ¼ retina * Local fine vitreous seeding may be present close to discrete tumor * Local subretinal seeding less than 3 mm (2DD) from the tumor
**Group D**	*Diffuse disease with significant vitreous or subretinal seeding*
	* Tumor(s) may be massive or diffuse * Subretinal fluid, present or past without seeding, involving up to total retinal detachment * Diffuse or massive vitreous disease may include “greasy” seeds or avascular tumor masses * Diffuse subretinal seeding may include subretinal plaques or tumor nodules
**Group E**	*Presence of any one or more of these poor prognosis features*
	* Tumor touching the lens * Tumor anterior to anterior vitreous face involving ciliary body or anterior segment * Diffuse infiltrating retinoblastoma * Neovascular glaucoma * Opaque media from hemorrhage * Tumor necrosis with aseptic orbital cellulites * Phthisis bulbi

**Table 3:** Study patients.

Group	I	II	III	B	C
**Total No. Eyes**	**7**	**6**	**6**	**19**	**11**
**Age at Dx, months (range)**	**3.14 (1–7)**	**7.67 (1–16)**	**10.5 (2–28)**	**7.21 (1–28)**	**5.9 (0–16)**
**(Enucleation or EBR)**	**0**	**0**	**0**	**0**	**1** *
**Mean follow up, months**	**20.8**	**17.4**	**7.6**	**17**	**15.2**
**Median follow up, months**	**25**	**15**	**9**	**13.5**	**13**
**Range of follow up, months**	**7 to 27**	**6 to 31**	**3 to 10**	**5 to 31**	**6 to 33**

* Eye enucleated after inability to repeat second IA treatment because of inability to cannulate the Ophthalmic artery.

Note: Because RE I, II and III have different definitions from ICRB B and C the numbers are not equivalent. The one “C” eye was RE Vb.

No patient developed metastatic disease and all patients are alive. No second cancers have developed.

Catheterization was successful in all patients except one. This patient was a 7 year-old girl with chromosome 13 deletion syndrome classified as ICRB C and RE Vb. The tumor had progressed despite 6 cycles of multi-agent systemic chemotherapy elsewhere and though the first attempt was successful the second treatment could not be done for technical reasons and the eye was enucleated. The child is alive and well.

**Figure 1:**
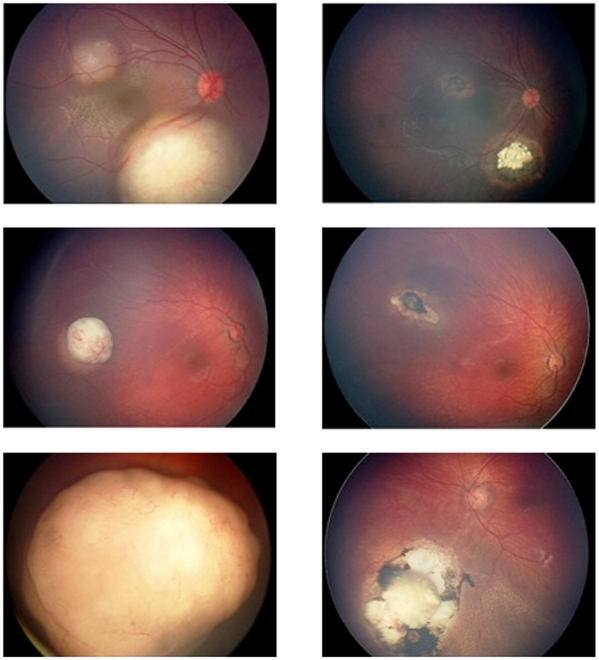
Digital fundus images of three retinoblastoma eyes before (left panel) and after (right panel) ophthalmic artery chemosurgery showing dramatic shrinkage of tumors without toxicity to the rest of the eye(s).

Ocular event-free survival (RE Group I, II and III) and patient survival were 100% at a median follow-up from the last treatment of 16 months (range 3–31 months). One Group C eye was enucleated after canulation was technically impossible on the second attempt. Fundus photographs of three eyes before and after treatment with ophthalmic artery chemosurgery are seen in Figure 1.

There were no seizures, strokes or hospitalizations after the procedure and no significant systemic toxicities. No child has required a port. Hematologic toxicity was graded by the standard CTCAEv4 scheme. Of 68 infusions 46 (67%) were associated with no hematologic toxicity. In 16 sessions grade III hematologic toxicity developed, in 6 sessions Grade IV toxicity developed. This includes one patient (previously treated elsewhere with multi-agent systemic chemotherapy 6 times) who developed Grade III ANC twice, Grade IV once and Grade IV platelet toxicity once and required hospitalization for febrile neutropenia.

Additional treatments were performed after intra-arterial chemotherapy in 11 of the 19 RE Group I-III eyes. Eight eyes (8/19; 42%) have not received additional treatments. Three eyes received both local cryotherapy and laser ablation and 8 eyes received laser ablation only. Regarding the ICRB Group B and C eyes, 19 of the 26 retained eyes were treated with focal laser only (15 eyes), cryotherapy only (1 eye), or both (3 eyes).

Compared with initial results, 30 Hz ERG responses as of the most recent follow-up examination were unchanged in 7 patients, improved by at least 25 µV in 7 patients, and decreased by at least 25 µV in 5 patients. Of those ERGs that deteriorated 4 remained in our “Good” category (>75 µV), which is considered a normal 30 Hz ERG response amplitude in most clinical laboratories.

## Discussion

Although ophthalmic artery chemosurgery was first performed just 5 years ago for childhood retinoblastoma it has rapidly become an accepted modality that saves eyes with advanced retinoblastoma that would otherwise have been enucleated. Unlike systemic chemotherapy single agent intrarterial chemotherapy can be curative alone. Because we had such success (and minimal toxicity) in advanced eyes we have now treated 19 RE and 30 ICRB eyes with less advanced intraocular disease. All patients survived and all treatable eyes were salvaged without the need for external beam irradiation (the only eye lost was one that could not be treated for technical reasons).

There are other ways to manage similar eyes. Some early staged disease can be managed with laser photocoagulation alone with success [12] but the conventional management for RE I-III was primary external beam irradiation until the 1990’s.

In the late 1960’s Cassady reported that 78.5% of RE I-III eyes could be salvaged with radiation [13]. In the 1970’s success rates of 80% were reported [14]. In the 1980’s success rates of 83% were reported [15]. In the 1990’s a success rate of 78.5% was reported in Groups I and II [16]. The largest report specifically on RE I-III treated with primary external beam irradiation was from our group [17]. This paper is also the only one to report specifically on radiation in RE I-III with information on ocular survival, need for additional treatment and patient survival (from metastases and second cancers). Overall, 85% of the eyes were salvaged though 53% needed additional treatments (such as laser or cryo). 4% of patients died of metastatic retinoblastoma and Kaplan-Meier estimates of second cancer incidence was 32% at 15 years.

Clinicians worldwide became progressively concerned about the risk of second cancers following radiation therapy and in the mid 1990’s switched to multi-agent systemic chemotherapy as primary treatment for retinoblastoma. More than 100 papers support the success of this approach in eyes with limited disease [18].

Systemic chemotherapy (usually Carboplatin, Vincristine and Etoposide) usually causes prompt reduction in size of intraocular tumors but because sustained regression is rare supplemental focal treatments with laser, cryotherapy, radioactive plaques and even external beam irradiation are routinely employed [19]. For example, Gündüz et al. noted that *no* RE I-III eye was controlled with chemotherapy alone and 23% did require subsequent external beam irradiation to salvage the eye [20]. A recent report from Korea emphasized that only 86% of REI-III eyes were salvaged using multi-agent systemic chemotherapy and these authors recommended 13 cycles of chemotherapy because of persistent tumor viability [21]. They also emphasized their concern about late re-growths in eyes treated with systemic chemotherapy and the propensity for chemotherapy related second malignancies.

Children with retinoblastoma have a high chance of long-term survival so complications from treatment are more than academic. As pointed out in an editorial in the Archives of Ophthalmology the overwhelming majority of papers (>90%) on the use of systemic chemotherapy (most written by ophthalmologists) for retinoblastoma include no information about side effects [22]. For those that report side effects complications such as the need for a port, transfusion, fever and neutropenia are common as is vincristine-related neurotoxicity [20]. Of further concern are recent reports highlighting late ototoxicity (as a result of carboplatin/cisplatin exposure) approaching 33% with carboplatin [23]–[25] and 100% with cisplatin [26]. Although all of the children are too young to measure the impact on fertility, recent articles have emphasized this potential toxicity of these drugs in the pediatric cancer population and urged clinicians to find alternatives [27].

Most distressing is the recognition that children with retinoblastoma who receive chemotherapy (epipodophyllotoxins and alkylating agents) are at risk for developing secondary leukemias [28]. The true incidence is unknown but more than 20 such children have been recognized worldwide. In addition, since some (up to 24%) of children with RE I-III receiving systemic chemotherapy will require subsequent external beam irradiation the concern for radiation-related second cancers has not disappeared with the use of primary systemic chemotherapy.

In conclusion, ophthalmic artery chemosurgery appears to be at least (if not more) effective than prior published series of similar eyes that were initially managed with primary radiation or systemic chemotherapy and is associated with excellent patient survival without the need for supplemental irradiation or the many unfortunate side effects of systemic chemotherapy. Many eyes achieve durable complete responses with single agent chemotherapy alone. Long-term data will be needed to confirm these encouraging findings.
